# A pleomorphic carcinoma of the lung producing multiple cytokines and forming a rapidly progressive mass-like opacity

**DOI:** 10.1186/1471-2407-14-588

**Published:** 2014-08-13

**Authors:** Masataka Matsumoto, Takashi Nakayama, Daiki Inoue, Kazufumi Takamatsu, Ryo Itotani, Manabu Ishitoko, Shinko Suzuki, Minoru Sakuramoto, Yoshiaki Yuba, Osamu Yoshie, Masaya Takemura, Motonari Fukui

**Affiliations:** Division of Respiratory Medicine, Kita-Harima Medical Center, Ono, Japan; Division of Respiratory Medicine, Kitano Hospital, Osaka, Japan; Division of Chemotherapy, Kinki University Faculty of Pharmacy, Osaka, Japan; Department of Pathology, Kitano Hospital, Osaka, Japan; Department of Microbiology, Kinki University School of Medicine, Osaka, Japan

**Keywords:** Lung cancer, FDG-PET, G-CSF, IL-6, IL-8, Neutrophilia

## Abstract

**Background:**

Lung cancer cells have been reported to produce cytokines, resulting in systemic reactions. There have been few reports showing that these cytokines induced the formation of an inflammatory mass around lung cancers.

**Case presentation:**

We encountered a patient with a pleomorphic carcinoma of the lung. This tumor produced interleukin (IL)-8, granulocyte colony-stimulating factor and IL-6, which in turn recruited inflammatory cells, such as CD8 positive lymphocytes, around the tumor, resulting in a rapidly growing tumor shadow.

**Conclusion:**

18 F-fluoro-deoxy-glucose positron emission tomography, in addition to a conventional radiological approach such as computed tomography, may detect immunological responses around a tumor.

## Background

Lung cancer cells have been reported to produce several cytokines and growth factors, especially granulocyte colony-stimulating factor (G-CSF), resulting in various systemic reactions [1–12]. We encountered a lung cancer patient with a peritumoral permeation shadow, which increased rapidly due to recruitment of cells by interleukin (IL)-8/CXCR1.

## Case presentation

**Figure 1:**
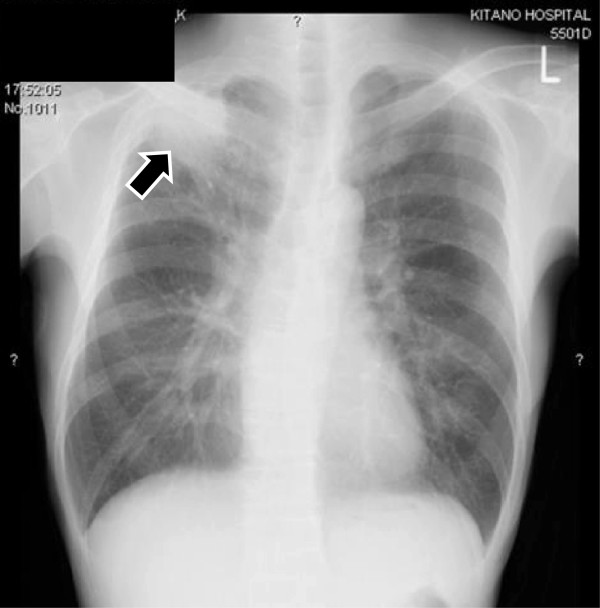
**Chest X ray on admission.** Arrow: tumor.

**Figure 2:**
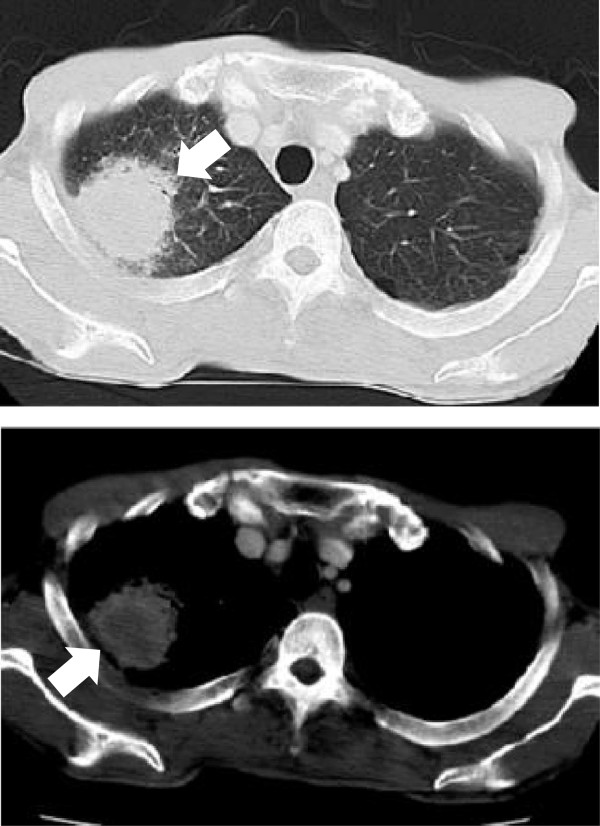
**Chest CT scan on admission.** Arrow: tumor.

A 52-year-old man complaining of a high-grade fever was referred to kitano hospital in August 2005. He previously had glucose intolerance, has a 26 pack-year history of smoking, and consumed 350 ml of beer per day. A chest X-ray showed a mass-like opacity in the apex of his right lung (Figure 1). A physical examination on admission showed reduced body weight (45.5 kg), sinus tachycardia (108/min), high respiratory rate (20/min) and high body temperature (38.0°C). Blood examination disclosed a marked leukocytosis (neutrophilia) and elevated serum concentrations of C-reactive protein (CRP) and liver enzymes (Table 1). A chest computed tomography (CT) scan showed a mass-like opacity in the apex of the right lung, with the inside of the mass being of low density (Figure 2).

**Table 1:** **Laboratory data on admission**

Hb 10.7 g/dl	BUN 10.5 mg/dl	*V.B*_*12*_ 1208 pg/ml
Plt 75.8x10^4^/μl	Cr 0.59 mg/dl	*Ferritin 1750 ng/ml*
*WBC* *36000/μ l*	T-pro 6.8 g/dl	*sIL-2R 2370 U/ml*
* NEUT* *89.0%*	Alb 2.7 g/dl	CEA 4.5 ng/ml
LYMPH 5.7%	*CRP 19.6 mg/dl*	CYFRA ≤1.0 ng/ml
MONO 4.1%	HbA_1c_ 6.0 g/dl	proGRP 14.8 pg/ml
EO 1.1%		NSE 4.5 ng/ml
BASO 0.1%	*IgG 2418 mg/dl*	*β*-D-glucan Negative
	*IgA 607 mg/dl*	*Mycoplasma* IgM Negative
*AST* *44 U/l*	IgM 85 mg/dl	*C. pneumoniae* IgM 0.95
*ALT 65 U/l*	*IgE 1103 IU/ml*	*C. Psittaci* Ab Negative
*LDH 1144 U/l*	C3 220 mg/dl	*Legionella* Ag in urine Negative
*ALP 1571 U/l*	C4 36 mg/dl	*Cryptococcus* Ag Negative
*γ-GTP 497 U/l*	CH_50_ 69 U/ml	
T-Bil 0.7 mg/dl		

The italics data was higher than normal level.

**Figure 3:**
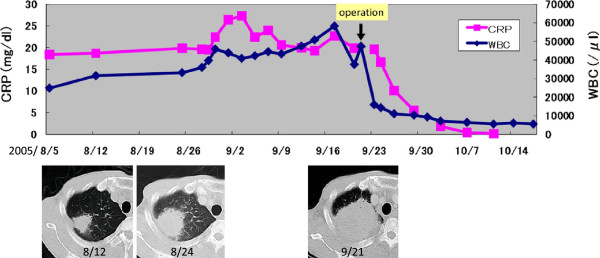
**Clinical course.** Before the operation, the mass-like opacity rapidly grew. After the operation, the patient’s neutrophilia and elevated CRP level rapidly subsided.

**Figure 4:**
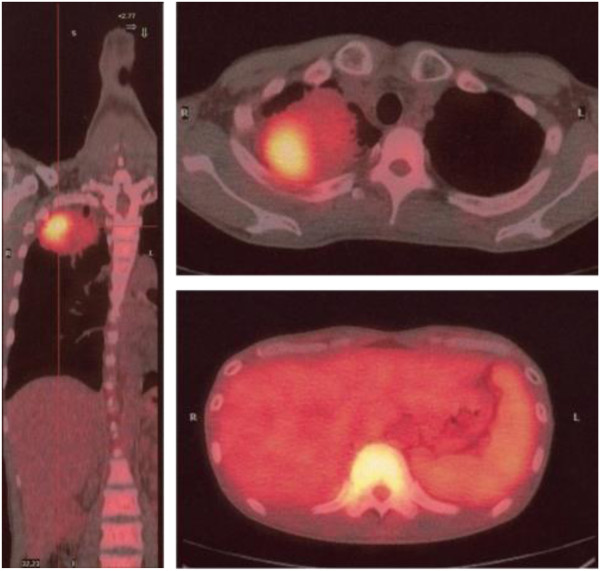
**FDG-PET/CT in September 2005.** FDG-PET/CT showed the localized uptake of FDG to the center of the mass-like lesion, in addition to diffuse uptake by the bone marrow and an enlarged liver and spleen.

**Figure 5:**
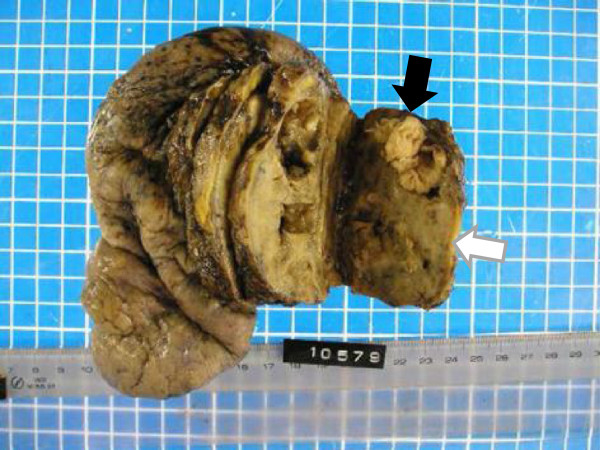
**Resected tumor.** Black arrow: main tumor including necrosis. White arrow: non-cancerous areas.

**Figure 6:**
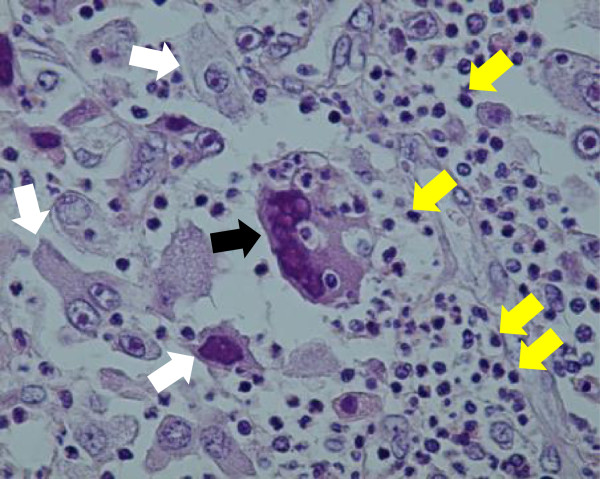
**Tumor tissue.** Black arrow: Giant cell, White arrows: Large cell carcinoma, Yellow arrows: Neutrophils.

His symptoms, blood examination and chest CT scan suggested a lung abscess. Despite the administration of antibiotics, however, the mass-like opacity rapidly grew (Figure 3). The transbronchial specimen and CT guided biopsy specimens were negative for bacteria, fungi and acid fast bacilli, but showed infiltration of lymphocytes. 18 F-fluoro-deoxy-glucose positron emission tomography (FDG-PET) showed the localized uptake of FDG to the center of the mass-like lesion and right hilar lymph node, in addition to diffuse uptake by the bone marrow and an enlarged liver and spleen (Figure 4). Before surgery, we considered that the lesion-like lung abscess was cancer and pneumonitis associated with cancer. The tumor was diagnosed as a cT3N1M0, stage IIIA lung cancer. Therefore, a right upper lobectomy was performed (Figures 5). The tumor was pathologically diagnosed as a pleomorphic carcinoma of the right upper lung (Figures 6). Following excision, it was shown to be of mixed subtype (predominantly large cell carcinoma with >10% giant cells and a few adenocarcinoma cells), measuring 40 × 34 × 28 mm, and graded pT3, pl0, G3, Ly0, V0, PLC0, R0, and pm0. The tumor was positive for lymph node metastases, and graded pN0, #2 (0/3), #3 (0/5), #4 (0/1), #10 (0/1), #11 (0/1), #12 (0/1) [pT3N0M0, pStage IIB]. The tumor was surrounded by intense infiltration of neutrophils and lymphocytes. The patient’s high grade fever, neutrophilia, and elevated CRP level rapidly subsided after the operation (Figure 3), as did his elevated serum concentrations of G-CSF and IL-6 (Table 2).

**Table 2:** **Cytokine concentrations before and after tumor resection**

	Before op	After op
IL-6 (pg/ml)	185	0.9
G-CSF (pg/ml)	235	<10

After the operation, elevated serum concentrations of IL-6 and G-CSF rapidly subsided.

Since the rapid growth of the mass-like shadow likely resulted from the infiltration of neutrophils and lymphocytes, we assessed the mechanisms underlying the infiltration of these cells.

## Materials & methods

### Lung cancer and control tissue

Frozen lung tissues containing cancerous and surrounding non-cancerous areas were obtained from this patient and from three other patients with lung cancer. All control tumors were adenocarcinomas. As the tumor and non tumor areas could be distinguished clearly, they were isolated macroscopically.

### Reverse transcription-polymerase chain reaction

Tissues samples were homogenized, and total RNAs were prepared using TRIzol reagent (Invitrogen Life Technologies, Carlsbad, CA, USA) and further purified using RNeasy Kit (Qiagen, Hilden, Germany). Total RNAs (1 μg) were reverse transcribed using oligo(dT)_18_ primer and SuperScript II reverse transcriptase (Invitrogen Life Technologies). The expression of mRNAs encoding chemokine receptors was assessed by reverse transcription-polymerase chain reaction (RT-PCR). First-strand complementary deoxyribonucleic acids (cDNAs), equivalent to 20 ng total RNA were amplified in a final volume of 20 μl containing 10 pmol of each primer [13, 14] and 1 U Ex-*Taq* polymerase (Takara Bio, Kyoto, Japan).

The amplification conditions consisted of an original denaturation at 94°C for 5 min, followed by 27 to 35 cycles of denaturation at 94°C for 30 sec, annealing at 60°C for 30 sec, and extension at 72°C for 30 sec, and a final extension at 72°C for 5 min. Chemokine cDNAs were amplified for 33 cycles, chemokine receptor cDNAs for 35 cycles, and glyceraldehyde-3-phosphate dehydrogenase (GAPDH) cDNA for 27 cycles. The amplification products (10 μl each) were electrophoresed on 2% agarose gels, which were stained with ethidium bromide.

### Immunohistochemical staining

Immunohistochemistry was performed using an automated immunostainer (Ventana BenchMark AutoStainer, Ventana Medical Systems, Tucson, AZ) with antibodies against CD4 (1:20, IF6, Novocastra), CD8 (1:20, 4B11, Novocastra), IL-8 (1:50, NYR-HIL8, Santa Cruz), IL-8R/CXCR1 (1:100, polyclonal, GeneTex, Inc.), G-CSF (1:100, FL-207, Santa Cruz) and IL-6 (1:20,000, polyclonal, R&D Systems). Neutrophils were recognized by morphological analysis.

This study was approved by the Kitano Hospital research ethics committee, and all subjects provided written informed consent.

## Results

### Analysis of chemokines and chemokine receptors

Because there are fewer chemokine receptors than chemokine ligands, we first assessed the levels of expression of mRNAs encoding all 18 types of chemokine receptors (CXCR1-6, CCR1-10, XCR1, and CX3CR1) in the inflammatory cells located around the tumor in our patient. RT-PCR showed that CXCR1 mRNA was strongly expressed in the non-cancerous area around the tumor.

We next assessed the expression in cancerous areas of mRNAs encoding several candidate chemokines, including IL-8/CXCL8, Mig/CXCL9, IP-10/CXCL10, I-TAC/CXCL11, CXCL16, MIP-1P-1 L16, MIP-1-α/CCL3, RANTES/CCL5, MCP3/CCL7, HCC-1/CCL14, HCC-2/CCL15, LEC/CCL16, MPIF-1/CCL23, MCP-1/CCL2, MCP-2/CCL8, MCP-4/CCL13, lymphotactin/XCL, and fractalkine/CX3CL. RT-PCR showed expression in the tumor of mRNA encoding IL-8, the ligand of CXCR1.

**Figure 7:**
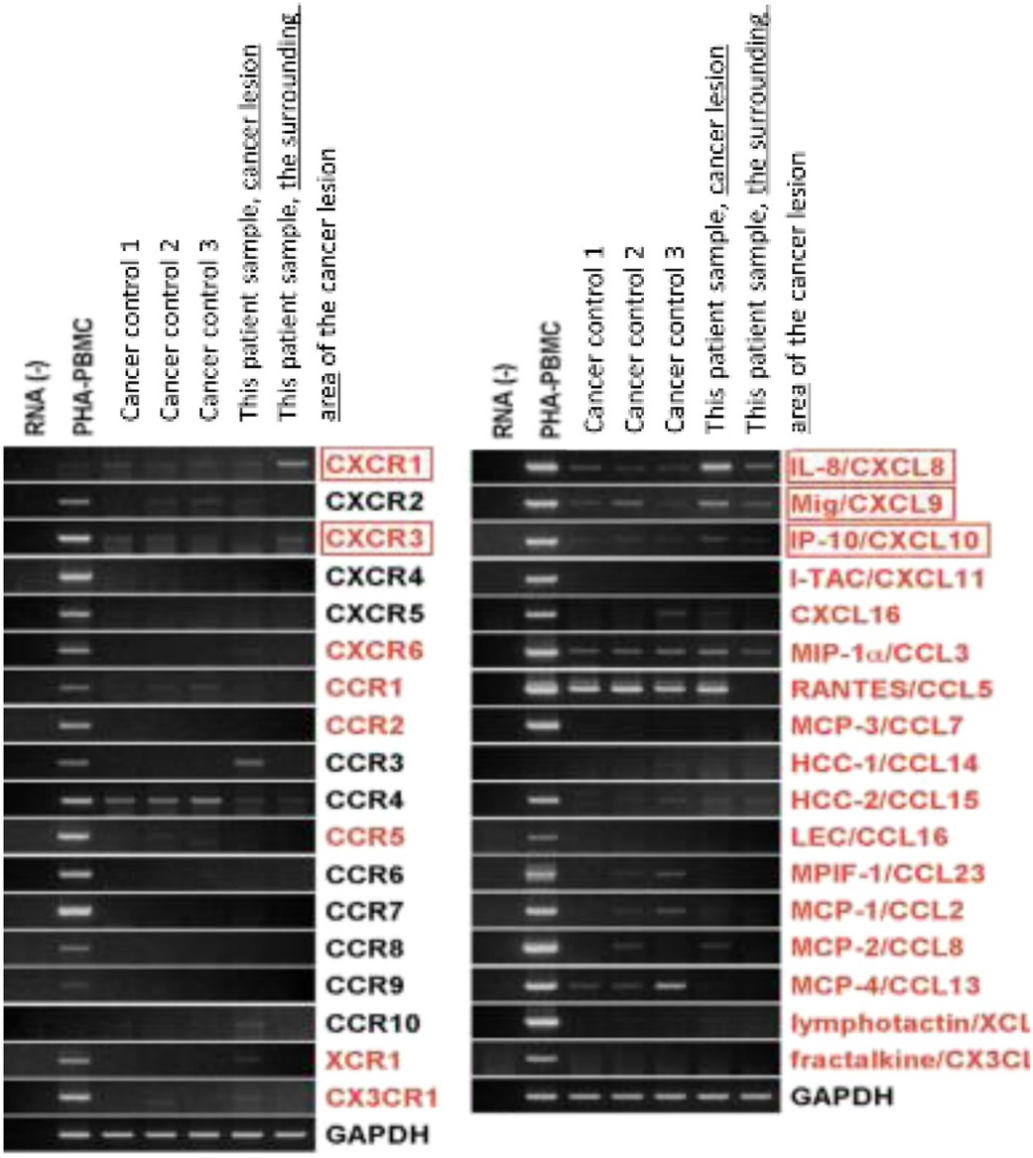
**RT-PCR analysis of chemokine receptors and their ligands.** RT-PCR showed that CXCR1 mRNA was strongly expressed in the surrounding area of the cancer and that mRNA encoding IL-8, the ligand of CXCR1, expressed in the cancer. In addition, CXCR3 mRNA was expressed in the cells surrounding area of the cancer, and that Mig and IP-10, the ligand of CXCR3, was expressed in the cancer itself. CCR3 was also expressed in the cancer.

In addition, we found that CXCR3 mRNA was expressed in the cells surrounding the tumor, and that Mig and IP-10, the ligand of CXCR3, was expressed in the tumor lesion itself. CCR3 was also expressed in the tumor, but we did not detect expression of its ligand (Figure 7).

### Immunohistological staining

The cancerous lesion in our patient was infiltrated by many CD8 positive lymphocytes and neutrophils and by a small number of CD4 positive lymphocytes. These lymphocytes were positively stained with anti-IL-8 antibody. The tumor cells were positive for G-CSF, partly positive for IL-8 and weakly positive for IL-6, but negative for IL-8R/CXCR1.

**Figure 8:**
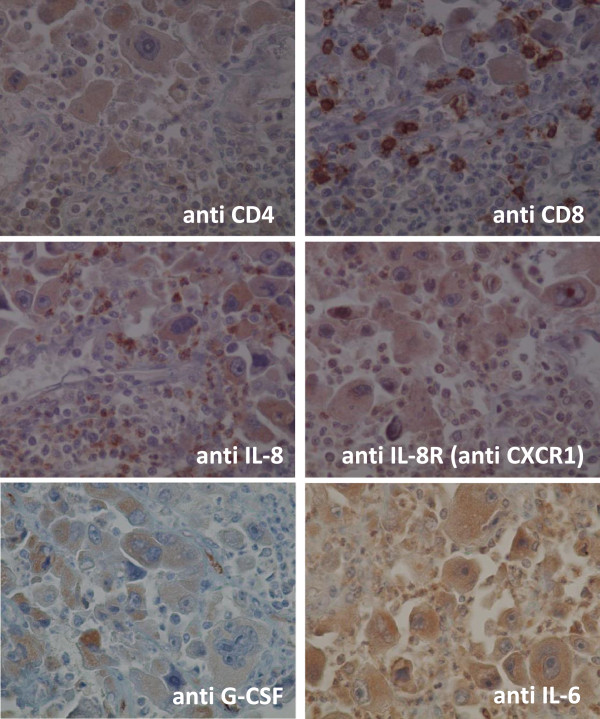
**Immunohistological staining of the cancer lesion (×400).** The cancerous lesion in our patient was infiltrated by many CD8 positive lymphocytes and neutrophils and by a small number of CD4 positive lymphocytes. These lymphocytes were positively stained with anti-IL-8 antibody. The tumor cells were positive for G-CSF, partly positive for IL-8 and weakly positive for IL-6, but negative for IL-8R/CXCR1.

**Figure 9:**
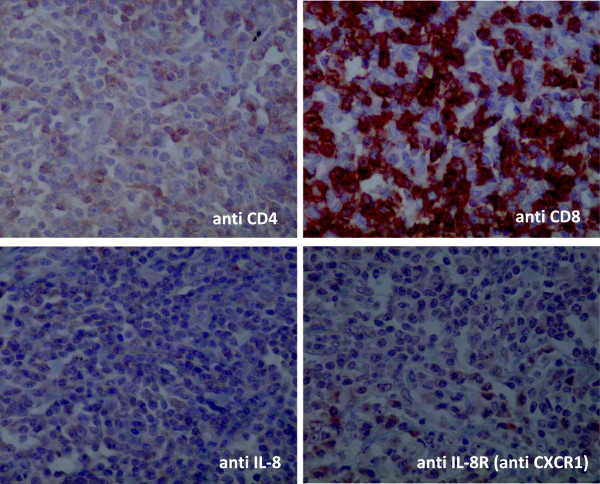
**Immunohistological staining of the tissue surrounding the cancer lesion (×400).** The area surrounding the tumor in our patient contained many CD8 positive lymphocytes, but few neutrophils. The neutrophils were negative for IL-8, whereas the infiltrating lymphocytes were weakly positive for IL-8R/CXCR1.

The area surrounding the tumor in our patient contained many CD8 positive lymphocytes, but few neutrophils. The neutrophils were negative for IL-8, whereas the infiltrating lymphocytes were weakly positive for IL-8R/CXCR1 (Figures 8 and 9).

## Discussion

We encountered a rare patient with a pleomorphic carcinoma and rapidly growing tumor shadow in the apex of the right lung. This patient presented with a high-grade fever and marked inflammatory responses. The excised tumor consisted of a carcinomatous lesion in the center, surrounded by the intense infiltration of inflammatory cells. FDG-PET showed that FDG uptake was localized to the center of the mass-like lesion, along with diffuse uptake in the bone marrow, liver and spleen.

In addition to high grade fever, neutrophilia, and elevated serum CRP concentration, our patient presented with elevated serum concentrations of G-CSF and IL-6. Following right upper lobectomy, however, these elevated concentrations became normalized. In addition, immunohistochemical examination showed that the cancer cells were positive for G-CSF and IL-6. The elevated IL-6 levels in this patient may have contributed to his high-grade fever and increased CRP levels [15], whereas the increased serum G-CSF levels may have contributed to neutrophilia and hematopoietic activation, as indicated by diffuse bone marrow uptake of FDG [1, 16]. These cytokines have been reportedly produced by lung cancers, especially by large cell carcinomas [2–11, 17]. Many lung cancers previously diagnosed as large cell carcinomas have been reclassified as pleomorphic carcinomas [18]. Additional studies are needed to determine the mechanism by which pleomorphic carcinomas produce cytokines.

The tumor shadow in this patient rapidly increased in size prior to surgery. Examination of the excised lung tissue showed that the area around the tumor consisted of non-cancerous tissue intensely infiltrated by lymphocytes and neutrophils. The formation of this inflammatory outer zone around the tumor was likely not due to the production by tumor cells of IL-6 or G-CSF. Rather, the tumor may have produced chemotactic factors, which in turn recruited the infiltration by inflammatory cells. To analyze this mechanism, we assessed the expression of several chemokines using RT-PCR and immunohistochemistry.

**Figure 10:**
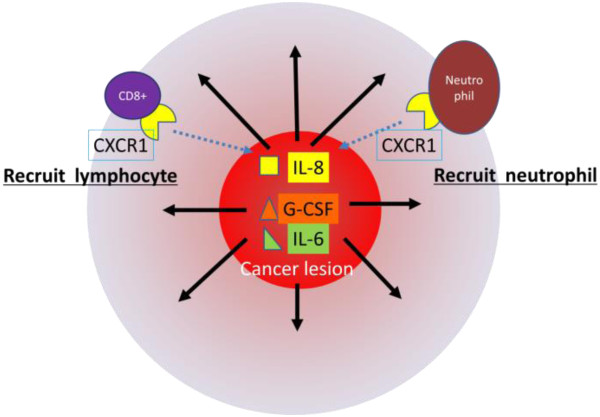
**Receptors and ligands in the cancer lesion and in the surrounding area.** Our findings suggest that the tumor cells produced IL-8, G-CSF and IL-6, IL-8 recruited CD8-positive lymphocytes and neutrophils from the bloodstream, resulting in the rapid organization of an inflammatory zone around the tumor.

In investigating the expression of chemokine receptors, we observed the intense expression of CXCR1 mRNA in the outer inflammatory zone. We also found that mRNA encoding IL-8 mRNA, the ligand of CXCR1 [19], was expressed by the tumor cells, and that these cells were partly stained with antibody to IL-8. Lymphocytes infiltrating the tumor were weakly positive for IL-8. In contrast, inflammatory cells in the area surrounding the tumor were negative for IL-8, while lymphocytes in these areas were weakly positive for IL-8R/CXCR1. Taken together, these findings suggest that the tumor cells produced IL-8, which recruited CD8-positive lymphocytes and neutrophils from the bloodstream, resulting in the rapid organization of an inflammatory zone around the tumor (Figure 10).

Although both CD8+ lymphocytes and neutrophils express the same IL-8R [20], CD8+ lymphocytes predominated around the tumor. This surrounding area was also positive for CXCR3, while mRNAs encoding Mig and IP-10, the ligands of CXCR3 and T-cell chemoattractants [20], were strongly expressed in the tumor itself. Because CD8 lymphocytes were recruited not only by IL-8 but also by Mig and IP-10, whereas neutrophils were recruited only by IL-8, these findings suggested that the imbalance between CD8 positive lymphocytes and neutrophils surrounding the tumor may be due to differences in the concentrations of these chemokines [20].

FDG-PET examinations have occasionally shown a pattern, in which FDG accumulates on the periphery of a tumor shadow, but not in the center of the lung tumor, owing to central necrosis and lung abscesses. The preoperative pattern of FDG uptake was unique in this patient, being localized to the center, but not the periphery, of the mass in the right lung apex. These finding suggest that FDG accumulated strongly in the cancerous lesion, but not in the outer inflammatory zone. Similar images on FDG-PET examination should suggest a tumor together with a local immunological response induced by the tumor.

## Conclusions

We encountered a patient with a pleomorphic carcinoma of the lung. This tumor produced several cytokines and chemokines, including G-CSF, IL-6, and IL-8, resulting in several systemic responses and a rapidly growing tumor surrounded by inflammatory cells. FDG-PET, in addition to a conventional radiological approach such as CT scanning, may distinguish between tumor-induced immunological responses and the tumor itself.

### Consent

Written informed consent was obtained from the patient for publication of this case report and any accompanying images.

## Abbreviations

IL: Interleukin
G-CSF: Granulocyte colony-stimulating factor
FDG-PET: 18 F-fluoro-deoxy-glucose positron emission tomography
CT: Computed tomography
CRP: C-reactive protein
mRNA: Messenger ribonucleic acid
RT-PCR: Reverse transcription-polymerase chain reaction
DNA: Deoxyribonucleic acid
GAPDH: Glyceraldehyde-3-phosphate dehydrogenase.

## References

[1] Hidaka D, Koshizuka H, Hiyama J, Nakatsubo S, Ikeda K, Hayashi A, Fujii A, Sawamoto R, Misumi Y, Miyagawa Y (2009). A case of lung cancer producing granulocyte colony-stimulating factor with a significantly high uptake in the bones observed by a FDG-PET scan. Nihon Kokyuki Gakkai Zasshi.

[2] Sukou N (2000). A case of large cell lung cancer with high level of G-CSF, IL-6, NSE. Haigan.

[3] Miyazawa M (1999). A case of large cell lung cancer producing G-CSF, IL-6. Jpn J Thorac Cardiovasc Surg.

[4] Umiwatari H (1997). About lung cancer producing Interleukin-6 and Granulocyte-colony stimulating factor at same time. Haigan.

[5] Sekido Y, Sato M, Usami N, Shigemitsu K, Mori S, Maeda O, Yokoi T, Hasegawa Y, Yoshioka H, Shimokata K (2002). Establishment of a large cell lung cancer cell line (Y-ML-1B) producing granulocyte colony-stimulating factor. Cancer Genet Cytogenet.

[6] Inoue M, Minami M, Fujii Y, Matsuda H, Shirakura R, Kido T (1997). Granulocyte colony-stimulating factor and interleukin-6-producing lung cancer cell line. LCAM. J Surg Oncol.

[7] Katsumata N, Eguchi K, Fukuda M, Yamamoto N, Ohe Y, Oshita F, Tamura T, Shinkai T, Saijo N (1996). Serum levels of cytokines in patients with untreated primary lung cancer. Clin Cancer Res.

[8] Tsuyuoka R, Takahashi T, Sasaki Y, Taniguchi Y, Fukumoto M, Suzuki A, Nakamura K, Kobayashi S, Kudo T, Nakao K (1994). Colony-stimulating factor-producing tumours: production of granulocyte colony-stimulating factor and interleukin-6 is secondary to interleukin-1 production. Eur J Cancer.

[9] Kimura H, Yamaguchi Y, Sun L, Iwagami S, Sugita K (1992). Establishment of large cell lung cancer cell lines secreting hematopoietic factors inducing leukocytosis and thrombocytosis. Jpn J Clin Oncol.

[10] Matsuguchi T, Okamura S, Kawasaki C, Shimoda K, Omori F, Hayashi S, Kimura N, Niho Y (1991). Constitutive production of granulocyte colony-stimulating factor and interleukin-6 by a human lung cancer cell line, KSNY: gene amplification and increased mRNA stability. Eur J Haematol.

[11] Suzuki A, Takahashi T, Okuno Y, Nakamura K, Tashiro H, Fukumoto M, Konaka Y, Imura H (1991). Analysis of abnormal expression of g-csf gene in a novel tumor cell line (KHC 287) elaborating G-CSF, IL-1 and IL-6 with co-amplification of c-myc and c-ki-ras. Int J Cancer.

[12] Wakimoto N (2010). Granulocyte colony-stimulating factor (G-CSF). Nihon Rinsho.

[13] Nakayama T, Hieshima K, Nagakubo D, Sato E, Nakayama M, Kawa K, Yoshie O (2004). Selective induction of Th2-attracting chemokines CCL17 and CCL22 in human B cells by latent membrane protein 1 of Epstein-Barr virus. J Virol.

[14] Nakayama T, Fujisawa R, Izawa D, Hieshima K, Takada K, Yoshie O (2002). Human B cells immortalized with Epstein-Barr virus upregulate CCR6 and CCR10 and downregulate CXCR4 and CXCR5. J Virol.

[15] Guo Y, Xu F, Lu T, Duan Z, Zhang Z (2012). Interleukin-6 signaling pathway in targeted therapy for cancer. Cancer Treat Rev.

[16] Mabuchi S, Morimoto A, Fujita M, Isohashi K, Kimura T (2012). G-CSF induces focal intense bone marrow FDG uptake mimicking multiple bone metastases from uterine cervical cancer: a case report and review of the literature. Eur J Gynaecol Oncol.

[17] Inoue M (1997). Analysis and establish of lung cell line producing G-CSF and IL-6, that have autocrine loop promoting cell proliferation. Haigan.

[18] Travis WD, Colby TV, Corrin B, Shimosato Y, Brambilla E (1999). Histological Typing of Lung and Pleural Tumours.

[19] Murphy PM (1997). Neutrophil receptors for interleukin-8 and related CXC chemokines. Semin Hematol.

[20] Kasakura S, Kouji M (2004). Cytokines and Chemokines.

[CR21] The pre-publication history for this paper can be accessed here:http://www.biomedcentral.com/1471-2407/14/588/prepub

